# Equilibrium, Thermodynamic, Reuse, and Selectivity Studies for the Bioadsorption of Lanthanum onto Sericin/Alginate/Poly(vinyl alcohol) Particles

**DOI:** 10.3390/polym13040623

**Published:** 2021-02-19

**Authors:** Talles Barcelos da Costa, Meuris Gurgel Carlos da Silva, Melissa Gurgel Adeodato Vieira

**Affiliations:** School of Chemical Engineering, University of Campinas, Albert Einstein Avenue, 13083-852 Campinas, Brazil; tallesbarcelos@hotmail.com (T.B.d.C.); meuris@unicamp.br (M.G.C.d.S.)

**Keywords:** alginate, sericin, poly(vinyl alcohol), lanthanum, bioadsorption

## Abstract

In a scenario of high demand, low availability, and high economic value, the recovery of rare-earth metals from wastewater is economically and environmentally attractive. Bioadsorption is a promising method as it offers simple design and operation. The aim of this study was to investigate lanthanum bioadsorption using a polymeric bioadsorbent of sericin/alginate/poly(vinyl alcohol)-based biocomposite. Batch system assays were performed to evaluate the equilibrium, thermodynamics, regeneration, and selectivity of bioadsorption. The maximum capture amount of lanthanum at equilibrium was 0.644 mmol/g at 328 K. The experimental equilibrium data were better fitted by Langmuir and Dubinin–Radushkevich isotherms. Ion exchange mechanism between calcium and lanthanum (2:3 ratio) was confirmed by bioadsorption isotherms. Thermodynamic quantities showed that the process of lanthanum bioadsorption was spontaneous (−17.586, −19.244, and −20.902 kJ/mol), endothermic (+15.372 kJ/mol), and governed by entropic changes (+110.543 J/mol·K). The reusability of particles was achieved using 0.1 mol/L HNO_3_/Ca(NO_3_)_2_ solution for up to five regeneration cycles. The bioadsorbent selectivity followed the order of lanthanum > cadmium > zinc > nickel. Additionally, characterization of the biocomposite prior to and post lanthanum bioadsorption showed low porosity (9.95 and 12.35%), low specific surface area (0.054 and 0.019 m^2^/g), amorphous character, and thermal stability at temperatures up to 473 K. This study shows that sericin/ alginate/poly(vinyl alcohol)-based biocomposites are effective in the removal and recovery of lanthanum from water.

## 1. Introduction

Rare-earth metals (REMs) consist of scandium, yttrium, and 15 lanthanides, i.e., lanthanum–lutetium [1]. REMs are known as the “vitamins of the industry” [2]. These metals are found mainly in China (55 million tons), United States (13 million tons), India (3.1 million tons), Brazil (2.2 million tons), and Australia (2.1 million tons) [3,4]. China concentrates about 97% of the worldwide REM production [4,5]. Lanthanum is a relevant and reactive REM due to its optical, magnetic, and chemical properties [6,7]. The concentration of lanthanum found in the earth’s crust is only 0.002% [5]. Lanthanum is usually used in high-tech applications, such as batteries, catalysts, ceramics, glasses, and superalloys [7,8]. Moreover, large volumes of effluent containing lanthanum are produced in these activities every day. The recovery of lanthanum from secondary sources is advantageous from the perspective of the circular economy [9].

Traditional methods employed for separating REMs (including lanthanum) include precipitation [10], solvent extraction [11], filtration [12], and ion exchange [13]. Among these methods, bioadsorption is considered an alternative to traditional methods because it is eco-friendly, economical, selective, low cost, and highly efficient for REM recovery at low concentration [14,15]. According to Gadd [16], any kind of biological material has an affinity for organic and inorganic pollutants, meaning there is enormous bioadsorption potential within countless types of biomaterials. Bioadsorbents from biological materials include microbial biomass, seaweed, industrial waste, agricultural waste, and natural waste, among other materials (such as cellulose, alginate, chitosan, etc.) [17,18,19]. These bioadsorbents have been extensively investigated over the past few years for the removal and recovery of REMs in aqueous medium [1,14,15]. Recently, adsorbents of alginate-based composites have been applied for lanthanum adsorption, such as calcium alginate particles [20], alginate/chitosan gel particles [21], alginate/γ-glutamic acid/poly(vinyl alcohol) (PVA) [22], alginate-clay-poly(n-isopropylacrylamide) hydrogel [23], and magnetic alginate particles [24,25]. Among these approaches, natural polymeric particles based on sericin/alginate crosslinked with poly(vinyl alcohol) (SAPVA) are promising biocomposites for application in bioadsorption processes [26,27].

Sericin, present in the silkworm cocoon (*Bombyx mori*), is a globular protein with molecular weight ranging from 10 to 300 kDa [28]. This natural protein represents about 15–35% of the cocoon weight, and it surrounds the structural filaments of the fibroin fiber in successive layers [29]. Despite being a waste from the silk industry, sericin has promising properties, including easy blending and crosslinking with other polymers, such as alginate and PVA, which results in biocomposites with improved physical–chemical and structural properties [26,30,31]. Alginate is a biopolymer that occurs as a structural component of the cell wall of brown seaweeds [32]. This natural polymer has characteristics such as biodegradability, biocompatibility, and ability to form gels in the presence of divalent cation, such as calcium [33,34]. PVA is a synthetic polymer that has hydroxyl groups that facilitate covalent crosslinking with other polymers [35,36]. A crosslinking agent is responsible for forming crosslinks between polymer chains [37]. Therefore, PVA forms crosslinks between alginate chains, sericin chains, and sericin/alginate chains, which improves the physical–chemical and mechanical characteristics of the particles, including a decrease in their water solubility [26,27].

This work is motivated by the absence of research investigating the use of sericin for the production of particles based on polymeric biocomposite and their interactions in the formation of blends, such as SAPVA, to recover REMs, such as lanthanum, in aqueous solutions. The ease of forming blends and the ability to crosslink these materials are also important to justify the research. In this study, we evaluated the bioadsorption of lanthanum by SAPVA particles in a batch system. Bioadsorption performance of the polymeric particles was investigated by equilibrium, thermodynamic, reuse, and selectivity studies at different experimental conditions. Moreover, the properties of SAPVA particles prior to and post lanthanum bioadsorption were conferred by characterization methods such as mercury porosimetry, Brunauer–Emmett–Teller (BET), X-ray diffraction (XRD), and thermogravimetric/differential thermogravimetric (TG/DTG) analysis.

## 2. Materials and Methods

### 2.1. Preparation of Alginate and Sericin Particles Chemically Crosslinked with Poly(vinyl alcohol)

Silkworm cocoons, which were used as the sericin source, were provided by Bratac Silk Mills Company (Londrina, Paraná, Brazil). The bioadsorbent preparation was carried out according to the methodology described by Santos et al. [38]. The cocoons were cleaned, cut (~1.0 cm^2^), washed with deionized water (OS20LXE, Gehaka, São Paulo, Brazil), and dried (323 K for 24 h). Sericin extraction from the cocoon shells was carried out physically (1.0 bar at ~393 K for 40 min) using an autoclave (AV-18, Phoenix Luferco, Araraquara, Brazil). The sericin solution was separated from silk fiber by filtration, kept at ~298 K for 12 h, and frozen/thawed for 24 h for sericin fractionation. The concentration of high molar mass sericin was adjusted to 2.5% (m/V) by dilution.

The blend was prepared by addition of 2.0% (m/V) sodium alginate (molecular weight 155,000 g/mol) to the high molar mass sericin solution of 2.5% (m/V) [39]. The polymers were stirred by a dispersant (Ultra-turrax/T18, IKA, China) until complete alginate solubilization. Thereafter, the crosslinking agent PVA (87–90% hydrolyzed, molar weight distribution 30,000–70,000 g/mol) was added in the ratio of 0.5% (m/V) [26]. The mixture was mechanically stirred (715W, Fisatom, São Paulo, Brazil) for 1 h. The sericin/alginate/PVA blend that was formed was dripped using a peristaltic pump (Masterflex/77800-60, Cole-Parmer, Chicago, IL, USA) in aqueous solution of 6.4% (m/V) calcium (Ca(NO_3_)_2_·4H_2_O, 99%) under magnetic stirring (TE-0854, Tecnal, Piracicaba, Brazil). The particles that were produced were stirred in calcium solution for 24 h in a jar test, washed with deionized water, dried at 313 K, and thermally crosslinked at 373 K for 24 h.

The elementary composition and morphological characterization of SAPVA particles were obtained by energy-dispersive X-ray spectroscopy (EDX; 6070, LEO Electron Microscopy, Cambridge, UK) and scanning electron microscopy (SEM; Leo 440i, LEO Electron Microscopy, Oxford, UK) techniques, respectively. These techniques have already been published in the literature [36]. The EDX technique showed that besides oxygen (57.04%) and carbon (25.98%), SAPVA particles was composed of calcium (16.99%). This result was attributed to the exchange of sodium ions present in the structure of sodium alginate and calcium ions in the solution used for the particle production process. The SEM technique showed that these particles presented a homogeneous composition, spherical shape, and low surface roughness. Additionally, according to the Fourier transform infrared spectroscopy (FTIR; Nicolet 6700, Thermo Fisher Scientific, Madison, WI, USA) technique [27], nitrogen and hydrogen functional groups were present in the SAPVA particles.

### 2.2. Metallic Ion Concentration Quantification

Lanthanum concentration was quantified by UV-visible spectroscopy (UVmini-1240, Shimadzu, Kyoto, Japan) by means of the xylenol orange method at 575 nm (Mukherji [40], adapted by Brião et al. [41]). In this method, samples of 2 mL of the lanthanum solution were mixed with 3 mL of 4.5 × 10^−4^ mol/L xylenol orange solution (C_31_H_28_N_2_Na_4_O_13_S, 99%) and 5 mL of 0.1 mol/L buffer solution at pH = 5.6 (C_2_H_4_O_2_, 99.88% and CH_3_COONa·3H_2_O, 99%). Calcium, cadmium, zinc, and nickel concentrations were determined by atomic absorption spectroscopy (AA-7000, Shimadzu, Kyoto, Japan).

### 2.3. Bioadsorption Assays in Batch System

The different lanthanum solutions applied in the tests were prepared by dilutions of a stock solution. This stock solution was prepared by dissolving 5 g of lanthanum nitrate (La(NO_3_)_3_·6H_2_O, 99.9%) in 100 mL of deionized water. Based on the chemical equilibrium diagram presented in Figure S1 (see Supplementary Material), the pH of the lanthanum solutions was fixed at 5.0 ± 0.5 and adjusted with nitric acid (HNO_3_, 65%). The experimental conditions were based on previous studies reporting the bioadsorption of toxic [39,42], precious [26,38], and rare-earth [36,43] ions by sericin/alginate particles. The bioadsorption assays were conducted in triplicate, and Microsoft Excel^®^ 2019 was used to calculate standard deviation.

#### 2.3.1. Equilibrium Study

Bioadsorption isotherms for the SAPVA/La system were obtained from solutions of La^3+^ ions with different initial concentrations (0.048 to 10.727 mmol/L) in contact with the SAPVA particles (dosage of 10.0 g/L) for 24 h, the time necessary to ensure that the system reaches equilibrium. All tests were carried out under constant mechanical agitation (200 r/min) in a shaker (TE-4200, Tecnal, Piracicaba, Brazil), with the pH adjusted and controlled at 5.0 ± 0.5 under different temperature conditions (298, 313, and 328 K). Bioadsorption isotherms were conducted at three different temperatures to determine the thermodynamic quantities of the SAPVA/La system. To assess ion exchange equilibrium, the concentration of Ca^2+^ light ions was also measured at each experimental point. The amount of La^3+^ ion uptake and Ca^2+^ ion release at equilibrium was calculated by Equation (1).

(1) $$q_{e}=\frac{\left(C_{0}-C_{e}\right)\cdot V}{m}$$

where $C_{0}$ is the initial metallic ion concentration in solution (mmol/L), $C_{e}$ is the metallic ion concentration at equilibrium (mmol/L), $q_{e}$ is the amount of metallic ions under equilibrium conditions (mmol/g), V is the volume of La^3+^ ion solution (L), and m is the mass of SAPVA particles (g).

The isotherm models (Equations (6)–(10)) were fitted to the experimental equilibrium data using the OriginPro^®^ 2020b software. Additionally, the thermodynamic quantities (ΔH, ΔS, and ΔG) were obtained by Equations (11)–(15). The isosteric heat of bioadsorption for the SAPVA/La system was estimated by Equation (16). Additionally, the SAPVA/La system design was evaluated to obtain the mass of particles necessary to achieve certain lanthanum reduction efficiencies (Equation (21)).

#### 2.3.2. Regeneration Study

To evaluate the reuse of SAPVA particles, 3.0 g of the particles was treated with 300 mL of 0.974 mmol/L lanthanum solution at pH of 5.0 ± 0.5 by magnetic stirring at about 200 r/min at ~298 K for 270 min. The lanthanum-loaded SAPVA particles were washed and dried and then desorbed by 0.1 mol/L HNO_3_/Ca(NO_3_)_2_ solution for 270 min. Subsequently, the regenerated SAPVA particles were washed until the solution pH reached 5.0. The regenerated particles were applied in five consecutive bioadsorption/desorption cycles with the same procedure. The amount of La^3+^ ions eluted from the SAPVA particles (*q_el_*, mmol/g) and the elution efficiency (*%E*) in each regeneration cycle were determined by Equations (2) and (3), respectively.

(2) $$q_{el}=\frac{C_{el}\cdot V_{el}}{m}$$

(3) $$\%E=\left(\frac{q_{el}}{q_{e}}\right)\cdot 100$$

where $C_{el}$ is the La^3+^ ion concentration eluted (mmol/L), and $V_{el}$ is the eluent volume (L).

Additionally, the amount of La^3+^ ion capture and Ca^2+^ ion release at equilibrium in each cycle was calculated by Equation (1).

#### 2.3.3. Selectivity Study

Chemical equilibrium diagrams were obtained by Visual MINTEQ^®^ 3.0 software with the same concentration of lanthanum = cadmium (Cd(NO_3_)_2_·4H_2_O, 99.9%) = zinc (Zn(NO_3_)_2_·6H_2_O, 99.9%) = nickel (Ni(NO_3_)_2_·6H_2_O, 99.9%) = 1.0 mmol/L. The selectivity of SAPVA particles toward lanthanum (1.097 mmol/L) was tested in a bioadsorption experiment in the presence of toxic metals, such as cadmium (1.142 mmol/L), zinc (1.137 mmol/L), and nickel (1.113 mmol/L), using a bioadsorbent dosage of 10 g/L. The selectivity tests were performed under agitation at 200 r/min for 24 h at 298 K and the pH defined in the chemical equilibrium diagrams. The selectivity coefficient ($S_{La^{3+}/M^{2+}}$) and distribution coefficient ($K_{d}$, L/g) were defined by Equations (4) and (5), respectively.

(4) $$S_{La^{3+}/M^{2+}}=\frac{K_{d\left(La^{3+}\right)}}{K_{d\left(M^{2+}\right)}}$$

(5) $$K_{d}=\frac{q_{e}}{C_{e}}$$

### 2.4. Theory and Mathematical Modeling

#### 2.4.1. Isotherm Models

Adsorption isotherms describe the accumulation of adsorbate at a bioadsorbent material at equilibrium conditions and constant temperature [44]. Langmuir [45] suggested a model (Equation (6)) to describe an adsorption process in which the adsorbent material surface presents a constant number of available and identical sites, and adsorption in monolayer is assumed [44].

(6) $$q_{e}=\frac{q_{max}\cdot K_{L}\cdot C_{e}}{\left(1+K_{L}\cdot C_{e}\right)}$$

where $q_{max}$ is the maximum capture amount at equilibrium (mmol/g), and $K_{L}$ is the Langmuir equilibrium constant (L/mmol).

The separation factor ($R_{L}$) can be calculated by Equation (7).

(7) $$R_{L}=\frac{1}{\left(1+K_{L}\cdot C_{0}\right)}$$

The Freundlich model (Equation (8)) was originated to describe an adsorption process occurring at the surface of a heterogeneous adsorbent material [46].

(8) $$q_{e}=K_{F}\cdot C_{e}^{1/n}$$

where $K_{F}$ is the Freundlich constant ((mmol/g)/(l/mmol)^1/n^), and n is the empirical constant (dimensionless).

The Dubinin–Radushkevich model (Equation (9)) assumes a nonhomogeneous bioadsorbent surface and a varying adsorption potential [47,48]. In addition, the mean adsorption energy (E, J/mol) can be determined by Equation (10).

(9) $$q_{e}=q_{max}\cdot exp\left[-K_{DR}\cdot {\left(R\cdot T\cdot ln\left(1+1/C_{e}\right)\right)}^{2}\right]$$

(10) $$E=\frac{1}{\sqrt{2\cdot K_{DR}}}$$

where $K_{DR}$ is the constant related to adsorption energy (mol^2^/J^2^), T is the temperature (K), and R is the ideal gas constant (8.314 J/mol·K).

#### 2.4.2. Thermodynamic Study

The investigation of thermodynamic quantities, such as Gibbs energy (ΔG, kJ/mol), enthalpy (ΔH, kJ/mol), entropy (ΔS, J/mol·K), and isosteric heat ($\Delta H_{ST}$, kJ/mol), provides an insight into the bioadsorption process. In this study, the van’t Hoff equation (Equation (11)) was used to estimate the thermodynamic quantities involved at lanthanum bioadsorption by SAPVA particles.

(11) $$\ln K_{C}=-\frac{\Delta H}{R\cdot T}+\frac{\Delta S}{R}$$

where $K_{C}$ is the thermodynamic equilibrium constant (dimensionless). In this study, the constant was calculated by Henry’s law (Equation (12)). To make the Henry’s constant, $K_{H}$ (L/g), dimensionless, Milonjić [49] suggested multiplying the constant by 1000 to convert into $K_{C}$ (Equation (13)). The enthalpy and entropy of adsorption can be obtained from the slope and interception of the $lnK_{C}$ versus 1/T plot, respectively.

(12) $$q_{e}=K_{H}\cdot C_{e}$$

(13) $$K_{C}=1000\cdot K_{H}$$

The Gibbs energy and activation energy ($E_{a}$) can be obtained by Equations (14) and (15), respectively.

(14) $$\Delta G=\Delta H-T\cdot \Delta S=-R\cdot T\cdot lnK_{C}$$

(15) $$E_{a}=\Delta H+R\cdot T$$

The isosteric heat was determined by the Clausius–Clapeyron equation (Equation (16)) [50].

(16) $$\ln C_{e}=\frac{\Delta H_{ST}}{R}\left(\frac{1}{T}\right)+constant$$

#### 2.4.3. Calculation of *R^2^* and *AICc*

The mathematical model’s fitness for equilibrium data was evaluated according to the determination coefficient, *R*^2^ (Equation (17)), and the corrected Akaike information criteria, *AICc* (Equation (18)) [51].

(17) $$R^{2}=1-\frac{\sum_{i=1}^{N}{\left(q_{exp}-q_{calc}\right)}^{2}}{\sum_{i=1}^{N}{\left(q_{exp}-\bar{q}\right)}^{2}}$$

(18) $$AIC_{C}=N\cdot ln\left[\frac{\sum_{i=1}^{N}{\left(q_{exp}-q_{calc}\right)}^{2}}{N}\right]+2p+\frac{2p\left(p+1\right)}{N-p-1}$$

where N is the number of observations, $q_{exp}$ is the experimental capture amount (mmol/g), $q_{calc}$ is the capture amount obtained by the model (mmol/g), $\bar{q}$ is the overall average of experimental values (mmol/g), and p is the number of model parameters.

### 2.5. Characterization Methods

Bioadsorbent particles were characterized prior to and post lanthanum bioadsorption. Porous structure was obtained by mercury porosimetry (AutoPore IV 9500, Micromeritics Instrument Corporation, Norcross, GA, USA) under low (0.03 to 2.41 bar) to high (2.41 to 4136.85 bar) pressure levels. Specific surface area was examined by BET analysis (Quantachrome/NOVA1200e, Anton Paar, Germany), measuring the adsorption/desorption of nitrogen at −469 K. Crystallinity analysis was conducted by XRD technique (X’Pert-MPD, Philips Analytical X-ray, The Netherlands) under 40 kV voltage, 40 mA, and 0.02° step size. Thermal analysis was performed in a nitrogen atmosphere with temperature range of 303 to 1073 K, gas outflow of 50 mL/min, and rate of 278 K/min (DTG 60, Shimadzu, Kyoto, Japan).

## 3. Results and Discussion

### 3.1. Bioadsorption Equilibrium

**Figure 1:**
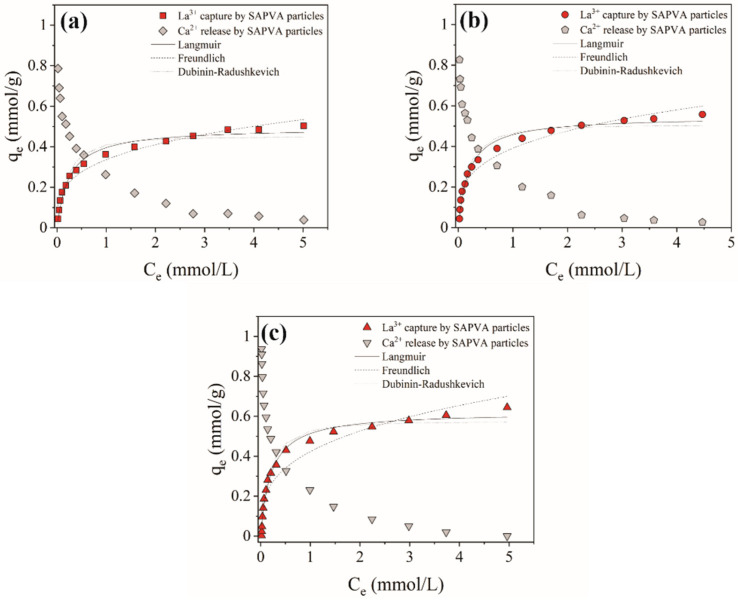
Experimental amounts of La^3+^ and Ca^2+^ ions and fit of isotherm models to lanthanum bioadsorption on sericin/alginate crosslinked with poly(vinyl alcohol) (SAPVA) particles at 298 (**a**), 313 (**b**), and 328 (**c**) K.

The equilibrium studies for lanthanum bioadsorption on the SAPVA particles were performed at temperatures of 298, 313, and 328 K. Figure 1 presents the experimental data and nonlinear fitting of the Langmuir, Freundlich, and Dubinin–Radushkevich models. Table 1 shows the parameters obtained by fitting the equilibrium models and statistical parameters (*R*^2^ and *AICc*). The average deviation values of the experimental isotherms were below 2.2%.

From Figure 1, it can be noted that the bioadsorptive lanthanum uptake isotherms showed favorable behavior. The maximum capture amount of lanthanum increased from 0.503 to 0.644 mmol/g with an increase in temperature from 298 to 328 K. This suggests that the lanthanum bioadsorption by SAPVA particles is endothermic and that chemical interactions may be present in the process. Santos et al. [38] and Costa et al. [36] also observed a similar behavior for gold and ytterbium bioadsorption in sericin/alginate particles crosslinked with proanthocyanidins and PVA, respectively.

According to Table 1, based on the values of *R*^2^ and *AICc*, it can be noted that more than one equilibrium model seemed to fit the experimental equilibrium data. Despite this, the Langmuir model showed a better fit at all temperatures, which was confirmed by the higher values of *R*^2^ and lower values of *AICc* values.

Langmuir model was developed considering that that the adsorbent surface has an energetically homogeneous sites available for monolayer adsorption [44,52]. On the other hand, the Dubinin–Radushkevich model does not consider an energetically homogeneous adsorbent surface [53]. The good fit for Langmuir and Dubinin–Radushkevich models was also reported by Andrade et al. [42], Santos et al. [38], and Costa et al. [36] for the bioadsorption of chromium, gold, and ytterbium by sericin/alginate particles, respectively. Additionally, the separation factor ($R_{L}$) obtained by the Langmuir model was between 0 and 1 for all temperatures, which indicates favorable lanthanum bioadsorption on the surface of SAPVA particles, corroborating the observed profile of the curves. The magnitude of mean adsorption energy (E) obtained by the Dubinin–Radushkevich model was less than 8 kJ/mol for all temperatures, which may be related to the presence of physical interactions in the SAPAS/La system [54]. Considering the complex nature of bioadsorbents, such as proteins and polysaccharides that contain long chains with distinct functional groups, the presence of homogeneous/heterogeneous sites and physical–chemical interactions is expected.

Table 2 shows information about the Langmuir maximum adsorption amount of different bio/adsorbents materials to lanthanum uptake. In general, the maximum bioadsorption amount of lanthanum obtained in this study was greater than most of the reported bio/adsorbents, which highlights the good performance achieved by the particles based on alginate, sericin, and PVA for the recovery of REMs in aqueous media.

Furthermore, the calcium content on the SAPVA particles was also determined for increasing La^3+^ ion concentrations. As presented in Figure 1, Ca^2+^ ions released from the SAPVA particles as La^3+^ ions were gradually bioadsorbed by them at all the temperatures evaluated. The uptake of La^3+^ ions by SAPVA particles was thus governed by the ion exchange mechanism. The maximum amount of Ca^2+^ released and La^3+^ captured increased from 0.786 to 0.936 mmol/g and 0.503 to 0.644 mmol/g with an increase in temperature from 298 to 328 K, respectively. This suggests an ion exchange process, where three Ca^2+^ ions were replaced by two La^3+^ ions at pH 5.0 at all the temperatures evaluated. The ion exchange reaction between SAPVA particles and La^3+^ ions can thus be written as Equation (19).

(19) $$2La^{3+}+3Ca{\left(SAPVA\right)}_{2}↔2La{\left(SAPVA\right)}_{3}+3Ca^{2+}$$

### 3.2. Thermodynamic Quantities

Table 3 presents the thermodynamic quantities (Δ*H*, Δ*S*, and Δ*G*) obtained for the SAPVA/La system. The linear plot of ln*K_d_* versus *1/T* is shown in Figure S2a (see Supplementary Material).

As shown in Table 3, it is possible to affirm that lanthanum capture by SAPVA particles was endothermic in nature (Δ*H* = +15.372 kJ/mol). Usually, in systems where adsorptive capacity increases with increasing temperature, the process is said to be endothermic, with absorption of heat from the surrounding binding the species to the solid [52]. Moreover, the magnitude of *∆H* below 80 kJ/mol indicates that interactions of a physical nature may be involved in the lanthanum bioadsorption onto SAPVA particles [69]. The positive Δ*S* (+110.543 J/mol·K) indicates that there was a high affinity between lanthanum and the SAPVA particles. The Δ*G* was negative at all temperatures (−17.586, −19.244, and −20.902 kJ/mol), which indicates that the capture of lanthanum by SAPVA particles occurred spontaneously.

Gao et al. [22], Tolba et al. [70], Elwakeel et al. [25], Iftekhar et al. [67], Cao et al. [2], and Cao et al. [71] found similar behavior of Δ*G* (negative), Δ*H* (positive), and Δ*S* (positive) for lanthanum bio/adsorption on alginate/γ-glutamic acid/poly(vinyl alcohol), poly(carboxymethyl) cellulose, magnetic alginate particles, gum Arabic-grafted polyacrylamide-based silica nanocomposite, polystyrene–poly(hydroxamic acid) copolymer, and poly(6-acryloylamino-hexyl hydroxamic acid) resin, respectively.

Figure S2b (see Supplementary Material) shows the isosteres obtained for lanthanum bioadsorption in SAPVA particles at different surface loading values (*q_e_* = 0.394, 0.438, and 0.476 mmol/g). Table 4 presents the isosteric heat found for each *q_e_* and the *R*^2^ coefficients of the linear fit. From Figure S2b, it can be noted that the isosteric heat of adsorption varied with the value of the surface loading. The isosteric heat decreased from 17.84 to 11.17 kJ/mol with an increase in the surface loading from 0.394 to 0.476 mmol/g. This behavior suggests that the SAPVA particles presented an energetically heterogeneous surface. Similar behavior was reported for propranolol bioadsorption in remaining biomass of algae [72], gold bioadsorption in sericin/alginate/proanthocyanidins particles [38], and ciprofloxacin adsorption in calcined Verde-lodo clay [73].

### 3.3. Simplified Batch Design

Bioadsorption isotherms can be used to predict the design of a batch bioadsorption system [36,38,72]. A scheme of a bioadsorption process in batch mode is shown in Figure S3 (see Supplementary Material). In the batch bioadsorption process, a volume (V) of lanthanum solution with an initial concentration ($C_{0}$) and a mass of SAPVA particles (m) with an initial amount of bioadsorbed lanthanum ($q_{0}$) are fed to the system. After the bioadsorption process, the lanthanum concentration is reduced from $C_{0}$ to $C_{1}$. The amount of bioadsorbed lanthanum changes from $q_{0}$ to $q_{1}$. Assuming that fresh SAPVA particles are used ($q_{0}=0$) and that the concentration of lanthanum removed from the fluid phase and captured by the particles is equal, the mass balance results in Equation (20).

(20) $$V\left(C_{0}-C_{1}\right)=m\left(q_{1}-q_{0}\right)=m\times q_{1}$$

In the case of the lanthanum bioadsorption on SAPVA particles, the Langmuir isotherm model gave the best fit to equilibrium data at 298 K. Thus, the $q_{1}$ value of Equation (20) was substituted by Equation (6), resulting in Equation (21).

(21) $$\frac{m}{V}=\frac{C_{0}-C_{e}}{q_{e}}=\frac{C_{0}-C_{e}}{\left(\frac{q_{max}\times K_{L}\times C_{e}}{1+K_{L}\times C_{e}}\right)}$$

**Figure 2:**
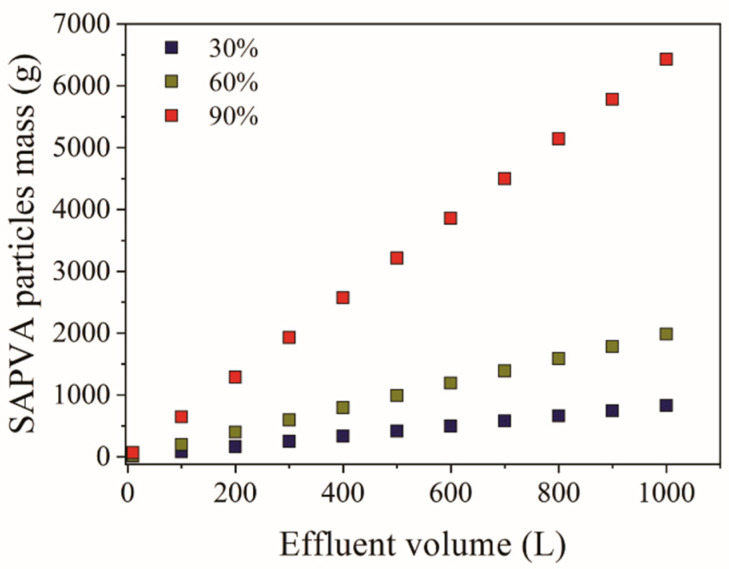
Simplified batch bioadsorption design for SAPVA/La system.

The simplified batch design for the SAPVA/La system was performed to obtain estimations of the amount of particles needed to achieve lanthanum reduction efficiencies of 30–90% from various volumes of lanthanum solutions at an initial concentration of 1.0 mmol/L. Figure 2 shows the batch bioadsorption design for the SAPVA/La system derived from Equation (21).

From Figure 2, it is possible to observe that the amount of SAPVA particles required increases as the volume of lanthanum solution increases to reach a certain removal efficiency. In other words, the greater the desired lanthanum removal efficiency, the higher the amount of SAPVA particles required to achieve this requirement. According to Figure 2, a lanthanum removal efficiency of 90% from 1000 L of lanthanum solution requires around 6500 g of particles. Therefore, the use of alternative bioadsorbents other than conventional activated carbon, such as natural polymeric particles based on sericin/alginate/PVA, can reduce the costs of the process because these particles are produced from a residue from silk manufacturing [31] and natural biopolymers extracted from brown seaweeds [74].

### 3.4. Regeneration Cycles

**Figure 3:**
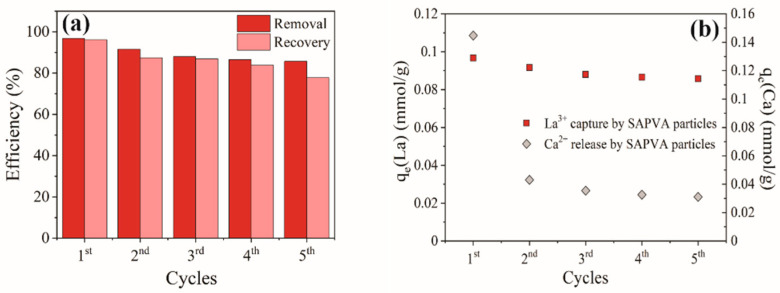
(**a**) Regeneration cycles of lanthanum onto bioadsorbent particles and (**b**) La^3+^ capture and Ca^2+^ release throughout reuse cycles.

Reusability of SAPVA particles was confirmed for up to five bioadsorption/desorption cycles using 0.1 mol/L HNO_3_/Ca(NO_3_)_2_ solution (Figure 3a). Bioadsorption and desorption efficiency was highest during the first cycle and decreased up to 85.68% (bioadsorption) and 77.70% (desorption) after the fifth cycle. The decrease in the capacity for reusing SAPVA particles during regeneration cycles may be related to the wear and shock between particles that damage their structure and reduce the number of bioadsorptive sites available.

The amount of Ca^2+^ released and La^3+^ captured in each bioadsorption cycle were obtained (Figure 3b). The amount of Ca^2+^ released and La^3+^ captured decreased from 0.097 to 0.086 mmol/g and 0.145 to 0.031 mmol/g during five consecutive reusability cycles, respectively. This result demonstrates that the Ca^2+^ ions did not completely disappear from the particles over the cycles. The addition of a Ca^2+^ ion source in the acid eluent increased competition between the H^+^, Ca^2+^, and La^3+^ ions for the bioadsorptive sites on SAPVA particles. Therefore, the presence of competing ions might have influenced the decreased bioadsorption amount toward La^3+^ ions over the cycles.

### 3.5. Selectivity

**Figure 4:**
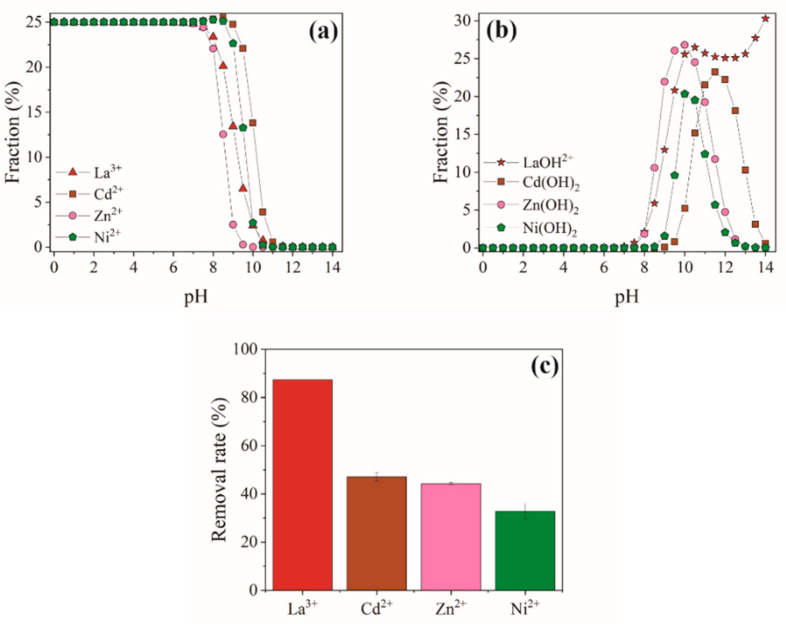
Bioadsorption selectivity over SAPVA particles: (**a**,**b**) distribution of metallic species as a function of pH and (**c**) effect of coexisting ions on the removal rate of lanthanum by SAPVA particles.

Considering that lanthanum is found together with other inorganic contaminants, such as zinc, nickel, and cadmium [75], the selectivity evaluation of SAPVA particles for lanthanum is important for real applications. From Figure 4a,b, it can be observed that lanthanum, zinc, nickel, and cadmium are present in their soluble ionic form (La^3+^, Zn^2+^, Ni^2+^, and Cd^2+^, respectively) at pH less than 7.5. Above this pH value, toxic metals precipitate in the form of Zn(OH)_2_, Ni(OH)_2_, and Cd(OH)_2_, besides the hydrolysis and formation of LaOH^2+^ species. Thus, to assess the bioadsorption selectivity of SAPVA particles, the pH of the mixture was adjusted at 5.0 ± 0.5. In this pH range, it is safe to state that only the soluble ionic species of metal ions are present.

Figure 4c shows the effect of coexisting ions on the removal rate of lanthanum. SAPVA particles bioadsorbed the lanthanum most easily (87.29 ± 0.01%), followed by cadmium (47.08 ± 1.86%), zinc (44.24 ± 0.57%), and nickel (32.85 ± 3.20%). In an ion exchange mechanism, metal ions with greater valence can be exchanged more expressively because adsorptive affinity depends mainly on the valence electronic structure and hydrated ionic radii [76]. Hence, toward divalent cations, these SAPVA particles show good selectivity toward trivalent lanthanum. Similar behavior was reported by Wang et al. [77] and Wang et al. [78] for neodymium capture in alginate/silica particles and alginate/γ-glutamic acid particles, respectively. Additionally, SAPVA particles showed great distribution coefficient and higher selectivity coefficient for La^3+^ ions (Table 5). These results show that SAPVA particles have good selectivity for lanthanum bioadsorption and future application in real effluents.

### 3.6. Biocomposite Characterization

**Figure 5:**
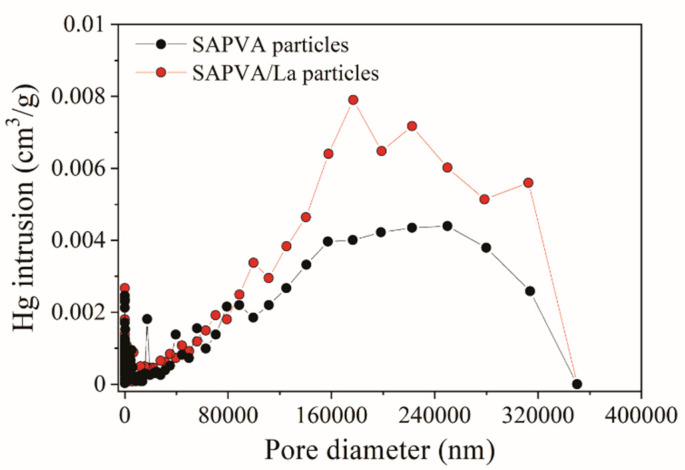
Pore size distribution profiles for SAPVA and SAPVA/La particles.

Apparent density and pore size distribution provided by the mercury porosimetry technique considers the real volume of the solid and the volume occupied by mercury in the bead’s pores. Figure 5 presents the pore size distribution in the SAPVA particles before and after lanthanum bioadsorption as a function of the mercury intrusion increment.

From Figure 5, the profiles obtained for the SAPVA and SAPVA/La particles reveal that the increase in mercury intrusion volume occurred mainly in regions whose pores were greater than 50 nm, which classifies the particles as macroporous [79]. The increase in mercury intrusion on the SAPVA/La particles might be associated with the particle solubilization that occurred during the bioadsorptive process. In fact, Santos et al. [26] reported that SAPVA particles showed a solubility of 3.74% in aqueous solution. The apparent densities were 1.341 and 1.282 g/cm^3^ for SAPVA and SAPVA/La particles, respectively. The reduction in apparent density indicates that lanthanum was captured on the surface of SAPVA particles. Additionally, there was an increase in the porosity of bioadsorbent particles after lanthanum capture from 9.95 to 12.35%, which is related to the bead’s solubility in water.

**Figure 6:**
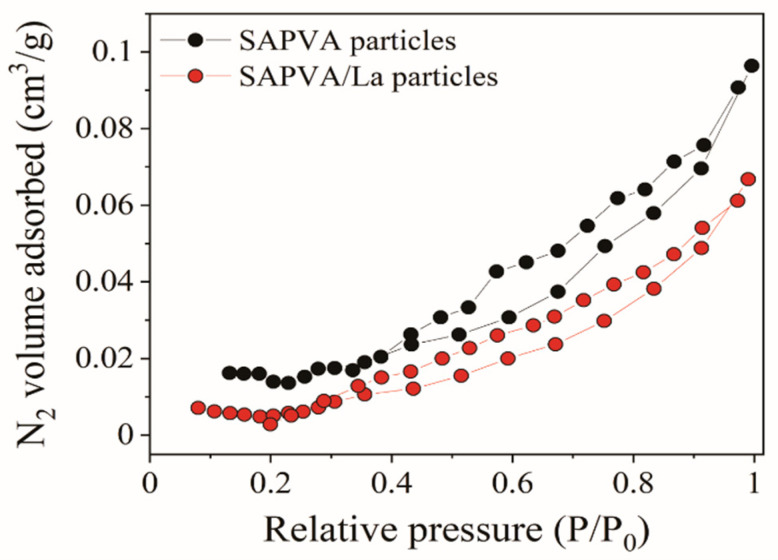
Nitrogen physisorption isotherms for SAPVA and SAPVA/La particles.

Structural features of the SAPVA and SAPVA/La particles were examined by nitrogen adsorption/desorption isotherms (Figure 6). As can be seen from Figure 6, both isotherms presented the same linear adsorption/desorption rates. Nitrogen isotherms of the SAPVA particles before and after lanthanum capture are classified as type II [80]. These results demonstrate that the porous structure of the bioadsorbent particles was mainly macroporous. This finding confirmed the mercury porosimetry technique that predicted this characteristic. Additionally, from the BET method, the specific surface area was calculated as 0.054 and 0.019 m^2^/g for SAPVA and SAPVA/La particles, respectively.

**Figure 7:**
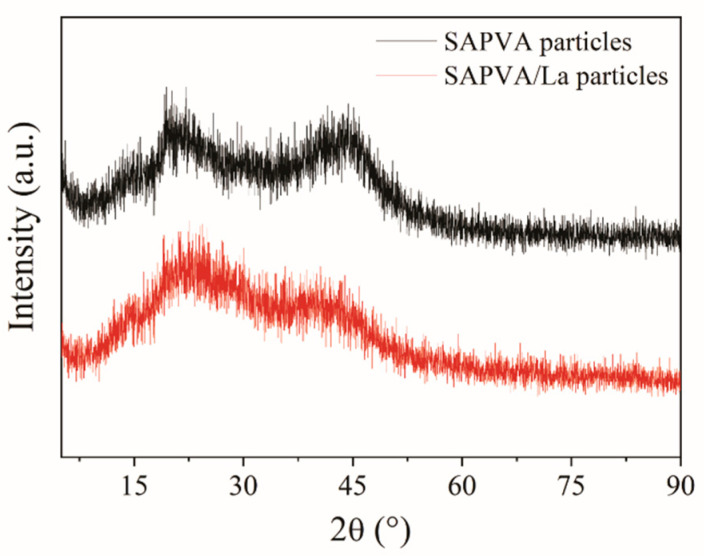
XRD spectra for SAPVA and SAPVA/La particles.

Figure 7 displays the diffractograms obtained for SAPVA and SAPVA/La particles. The similarity between the XRD spectra obtained for SAPVA particles preprocess and loaded with lanthanum suggests that the lanthanum bioadsorption process made little change to the crystalline structure of the bioadsorbent. In both diffractograms, a characteristic peak of sericin β-sheets structure is observed around 2θ = 19.2° and a low intensity peak at 2θ = 43° [42,81]. Therefore, it can be concluded that even after bioadsorption process, these particles showed a predominantly amorphous nature.

**Figure 8:**
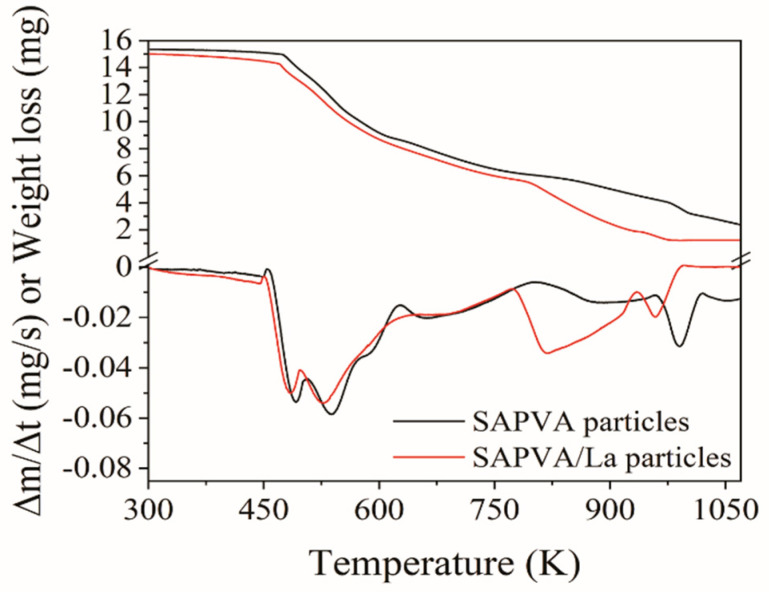
Thermal analysis curves for SAPVA and SAPVA/La particles.

The thermal stability of the particles before and after lanthanum bioadsorption was evaluated by thermogravimetric analysis. Figure 8 shows the thermograms for SAPVA and SAPVA/La particles. The weight loss up to 473 K for both particles is related to water loss. In the range between 473 and 673 K, the sudden weight loss is related to the degradation of amine groups, cleavage of the peptide bonds, and degradation of sericin [82]. Besides, between 473 and 673 K, alginate and PVA degradation begins [36,83,84]. The weight loss between 673 and 873 K can be attributed to the continued degradation of SAPVA and SAPVA/La particles. For the region between 873 and 1073 K, there was a great weight loss, which may be associated with the decomposition of the components of bioadsorbent particles. Regarding the weight loss percentage, values of 84.37 and 91.76% were obtained for SAPVA and SAPVA/La particles, respectively. This result indicates that there was a decrease in the thermal stability of the particles after lanthanum bioadsorption.

### 3.7. Practical Applications and Future Research Perspectives

Due to the current scenario, the recovery of REMs, such as lanthanum, from effluents is a matter of extreme importance for technological and environmental purposes [1]. In this work, sericin/ alginate/poly(vinyl alcohol)-based biocomposite particles were examined for the removal and recovery of lanthanum from aqueous medium. The use of the waste protein obtained from the textile industry during processing of silk yarns and alginate extracted from brown seaweed, which is widely available off the Brazilian coast, is advantageous because the costs involved are low and this biomass is naturally abundant and renewable [31,85,86].

The results of this work show that besides all the advantages, the commercial application of SAPVA particles for removal and recovery of lanthanum is also faced with several challenges that need to be addressed before scaling up this technology. Lanthanum removal from aqueous medium by SAPVA particles is limited in successive regeneration cycles and selective bioadsorption of lanthanum. Despite this, the maximum capture amount of lanthanum by SAPVA particles in this work (0.644 mmol/g) is greater than most of the reported bio/adsorbents (Table 2). Therefore, to improve reuse, selectivity, and bioadsorption amount of this system, techniques for the functionalization and modification of SAPVA particles are necessary. These techniques include organic and inorganic modifications or thermal treatments to obtain SAPVA particles with greater bioadsorption capacity, selectivity, and stability for reuse. In addition, continuous bioadsorption assays on a fixed-bed column are important for scale-up. Finally, future studies should be focused on investigation of the potential of SAPVA particles toward lanthanum and other REM and non-REM removal from real effluents.

## 4. Conclusions

The present study shows that sericin/alginate/poly(vinyl alcohol)-based biocomposite particles are efficient bioadsorbents for lanthanum capture. The maximum amount of lanthanum captured (0.644 mmol/g) and calcium released (0.936 mmol/g) at equilibrium were obtained at 328 K. Thermodynamic quantities revealed that the lanthanum capture by SAPVA particles was endothermic and spontaneous. Reuse cycles in batch system proved that the produced biocomposite particles could be used as recyclable bioadsorbents. SAPVA particles also showed good selectivity for lanthanum bioadsorption from the mixture with cadmium, zinc, and nickel. Characterization techniques showed that the SAPVA particles (before and after lanthanum capture) had low porosity and low specific surface area and were amorphous, thermally stable, and macroporous. Therefore, enhancement of the bioadsorption capacity, reuse, and selectivity of SAPVA particles through functionalization and modification techniques and its application in real effluents are recommended for future studies.

## Figures and Tables

**Table 1:** Equilibrium isotherm parameters of lanthanum bioadsorption onto SAPVA particles obtained at 298, 313, and 328 K.

Models	Parameters	Temperature		
		298 K	313 K	328 K
Experimental	*q_max_* (mmol/g)	0.503	0.557	0.644
Langmuir	*q_max_* (mmol/g)	0.495	0.548	0.621
	*k_L_* (L/mmol)	3.950	5.011	4.762
	*R_L_*	0.025	0.019	0.020
	*R* ^2^	0.984	0.984	0.984
	*AICc*	−121.628	−117.638	−113.298
Freundlich	*n*	0.328	0.382	0.423
	*R* ^2^	3.184	3.203	3.183
	*AICc*	0.949	0.931	0.915
	*k_F_* [(mmol/g)·(L/mmol)^1/n^]	−101.268	−92.426	−85.024
Dubinin– Radushkevich	*q_m_* (mmol/g)	0.450	0.507	0.574
	*k_DR_* (mol²/J^2^)	3.23 × 10^−8^	2.50 × 10^−8^	2.41 × 10^−8^
	*E* (kJ/mol)	3.937	4.472	4.557
	*R* ^2^	0.964	0.973	0.977
	*AICc*	−107.119	−108.465	−107.041

**Table 2:** Langmuir maximum bio/adsorption amount for lanthanum capture by different materials.

Bio/Adsorbents	*q_max_* (mmol/g)	References
*Platanus orientalis* leaf powder	0.206 *	Sert et al. [55]
Magnetic GMZ bentonite	0.132 *	Wu et al. [56]
P204 immobilized magnetic GMZ bentonite	0.292 *	Chen et al. [57]
P507 functionalized magnetic	0.402 *	Wu et al. [58]
SnO_2_–TiO_2_ nanocomposites	0.488 *	Rahman et al. [59]
*Stichococcus bacillaris*	0.367 *	Birungi et al. [60]
*Chlorella vulgaris*	0.537 *	
Cys@CHI-magnetic	0.129 *	Galhoum et al. [61]
Kaolin	0.012 *	Xiao et al. [62]
EDTA-β-cyclodextrin	0.343	Zhao et al. [63]
GO nanosheets	0.617 *	Ashour et al. [64]
KMnO_4_–AC	0.001 *	Kano et al. [65]
CLN/SiO_2_	0.212 *	Iftekhar et al. [66]
GA-g-PAM/SiO_2_	0.057 *	Iftekhar et al. [67]
MIL-101-PMIDA	0.269 *	Lee et al. [68]
SAPVA particles	0.503	This study
	0.557	
	0.644	

* Converted from the unit of mg/g showed in the papers.

**Table 3:** Thermodynamic quantities of lanthanum bioadsorption on SAPVA particles.

*T* (K)	Δ*H* (kJ/mol)	Δ*S* (J/mol·K)	Δ*G* (kJ/mol)	*E_a_* (kJ/mol)
298	+15.372	+110.543	−17.586	17.851
313			−19.244	17.976
328			−20.902	18.101

**Table 4:** Isosteric heat values of lanthanum bioadsorption on SAPVA particles.

*q_e_* (mmol/g)	Δ*H_ST_* (kJ/mol)	*R* ^2^
0.394	17.84	0.999
0.438	12.63	0.981
0.476	11.17	0.981

**Table 5:** The values of $K_{d}$ and $S_{La^{3+}/M^{2+}}$ of coexisting ions.

Coexisting Ions	$K_{d}\;(L/g)$	$S_{La^{3+}/M^{2+}}$
La^3+^	0.686	-
Cd^2+^	0.089	7.702
Zn^2+^	0.079	8.655
Ni^2+^	0.049	13.987

## Supplementary Materials

The following are available online at https://www.mdpi.com/2073-4360/13/4/623/s1, Figure S1: Lanthanum ion species in aqueous solution simulated using Visual MINTEQ^®^ 3.0 software ($C_{0,La}$ = 11.0 mmol/L), Figure S2: Plot of $lnK_{d}$ versus 1/T (a) and plot of $lnC_{e}$ versus 1/T (b) obtained for bioadsorption of lanthanum by SAPVA particles, and Figure S3: Scheme of a bioadsorption process in batch mode.
