# Supplementary material for: Equilibrium, Thermodynamic, Reuse, and Selectivity Studies for the Bioadsorption of Lanthanum onto Sericin/Alginate/Poly(vinyl alcohol) Particles
Source: Polymers (Basel). 2021 Feb 19;13(4):623. doi: 10.3390/polym13040623 (PMC7922337; doi:10.3390/polym13040623)
Supplement: Supplementary file 1 [file polymers-13-00623-s001.pdf]

polymers

*Supplementary Material*

# Equilibrium, Thermodynamic, Reuse, and Selectivity Studies for the Bioadsorption of Lanthanum onto Seri-cin/Alginate/Poly(vinyl alcohol) Particles

**Talles Barcelos da Costa[a], Meuris Gurgel Carlos da Silva[a] and Melissa Gurgel Adeodato Vieira[a]***

[a]School of Chemical Engineering, University of Campinas, Albert Einstein Avenue, 13083-852, Campinas, São Paulo, Brazil; tallesbarcelos@hotmail.com (T.B.d.C.); meuris@unicamp.br (M.G.C.d.S.)
* Correspondence: Correspondence: melissag@unicamp.br; Tel: +55 19 3521-0358

**Summary.** This document contains three figures on three pages.

**Bioadsorption assays in batch system**

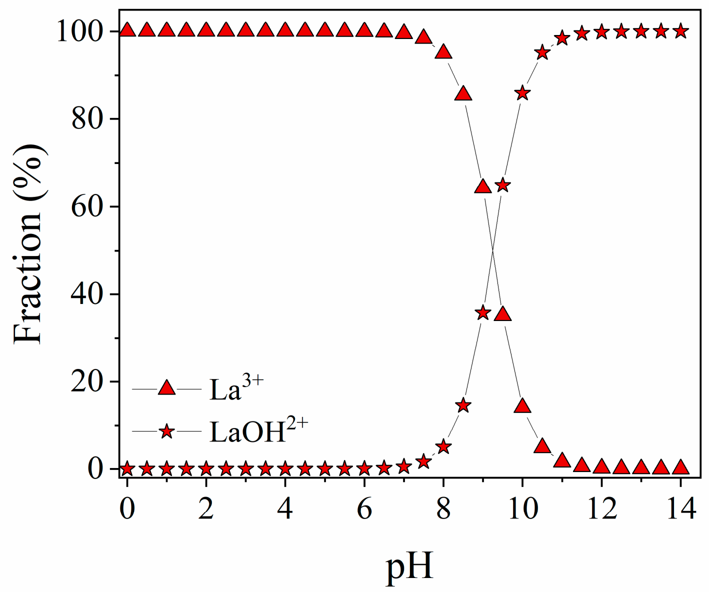


**Figure S1.** Lanthanum ions species in aqueous solution simulated using Visual MINTEQ® 3.0 software ($C_{0,La}$= 11.0 mmol/L).

**Thermodynamics quantities**

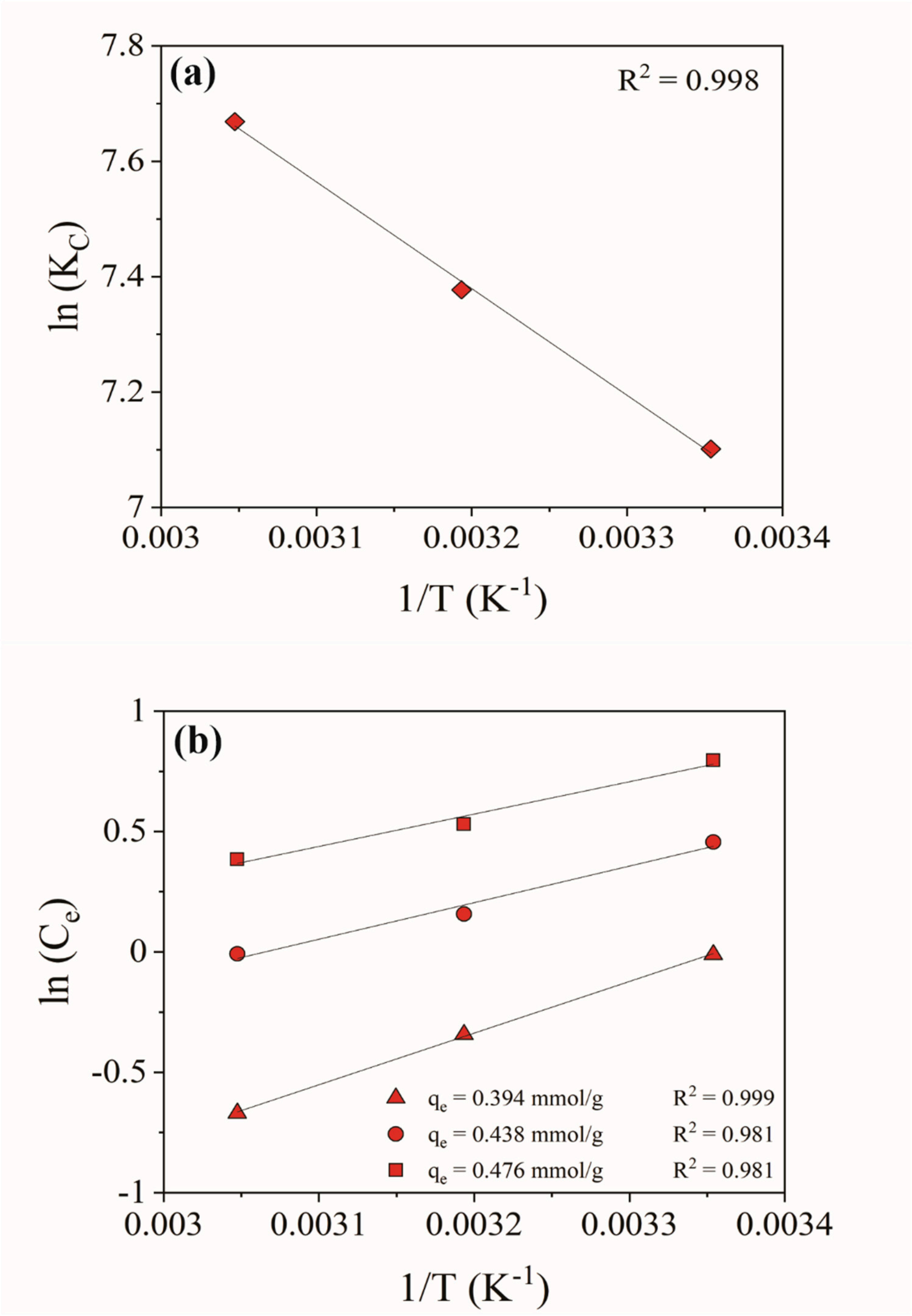


**Figure S2.** Plot of $lnK_d$ versus $1/T$ (a) and Plot of $lnC_e$ versus $1/T$ (b) obtained for bioadsorption of lanthanum by SAPVA particles.

**Simplified batch design**

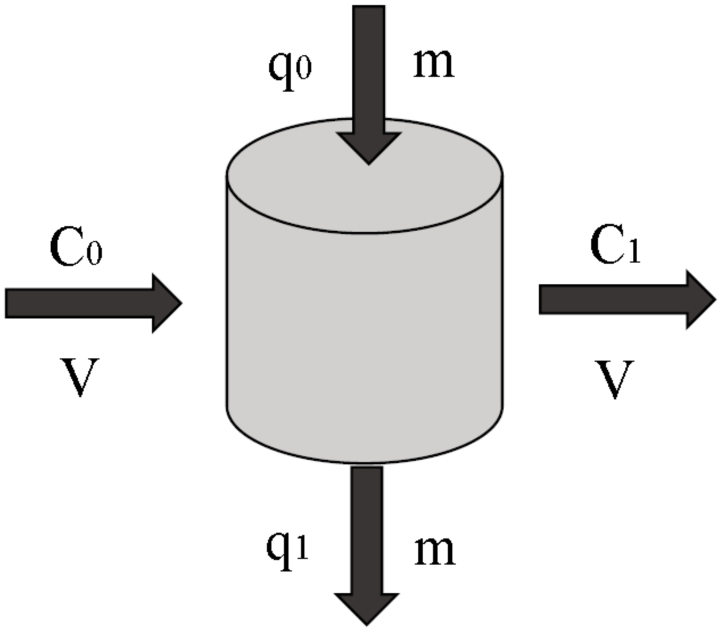


**Figure S3.** Scheme of a bioadsorption process in batch mode.
